# Penetrating Wounds of the Chest

**Published:** 1919-01

**Authors:** 


***Penetrating Wounds of the Chest.*** By Captain R. B. Blair and Major G. C. Shattuck, R. A. M. C. *Journal of the Royal Army Medical Corps*, September, 1918.

The authors insist upon the value of wise surgical intervention and the necessity of co-operation with a radiologist of experience. A series of observations made on cases admitted to a C. C. S. between July 31st and October 1st, 1917, are then recorded. The time that elapsed between wounding and admission of the patient varied, as a rule, between 6 and 24 hours, and the work was based upon the belief that all wounds of the chest should be considered from the standpoint of sepsis, and that early surgical intervention would prove an advantage.

*Operative Technic in General.* Operations of the chest must be performed under strict aseptic conditions. The operator is guided by the position of the wound and by the radiologist's findings with regard to the site of the operation. If the missile is situated in a portion of the lung or pleura accessible through the wound, the entrance wound should be excised. The hemothorax should then be evacuated, mopping it out by means of a long roll of gauze introduced on forceps. Finally the pleura is cleared of blood clots, and irrigated or swabbed as clean as possible before closure; the chest is then closed in layers, the first being a muscle pleural one. The skin, once sutured, is painted with picric acid solution and a dressing applied.

*Type Operations.* (1) Parietal : Complete excision of the wound in the parieties.

(2) Thoracotomy : Large resection of rib made either through the wound or through an uninjured part.

(3) Drainage : (a) Primary, when the infection has become established to a marked degree; (b) Secondary, for established infection which resists conservative treatment.

(4) Lesions of the diaphragm : Incision along the line of the 6th rib, division of the costal cartilage, the intercostal muscle, and the pleura. The diaphragm is sutured with catgut.

(5) Subscapular lesions : The scapula is displaced by dividing the rhomboids and serratus magnus with an incision extending the full length of the scapula. The scapula can then be moved so as to give access to the chest wall.

(6) Exposure of the pericardium : Division of the attachment of the rectus abdomini, of the fibres of the pectoralis and separation of the attachment of the intercostal muscles to both borders of the cartilage. The perichondrium is separated all round and the fibres of the triangularis sterni set aside. The pericardium is thus exposed and the pleural sinus can be reached.

*General indications for operation.*

(1) Lacerated wounds should be excised.

(2) Dirty or inflamed wounds of the chest wall should be completely excised and cleaned.

(3) Wounds with comminution of rib or scapula call for complete excision and removal of bone fragments.

(4) Open pneumothorax should be closed at the earliest possible moment by skin suture.

(5) Retention of a large missile in the parieties or thorax : in either case the foreign body should be removed and the chest closed.

(6) Wounds of the diaphragm should be carefully sutured.

(7) Infection of the pleural cavity : the chest should be closed, if only for a day, in order to permit the infection to become localized.

The time of operation and preliminary treatment vary according to cases. Rest in bed, warmth, and a dose of morphia to relieve pain are the principles generally resorted to; blood transfusion has, in a number of cases, proved valuable and might be used more often with advantage. Before operation the size and exact position of the missile should be carefully determined.

*Post-operative treatment* : The patient is placed in a semi-recumbent, later in a semi-sitting position. Fluids are allowed for the first 24 hours after operation. At the end of 48 hours, the chest should be needled, a specimen taken for examination, and aspiration performed if there are more than a few ounces of fluid; in cases when the latter is infected, the chest should be aspirated at intervals of from 24 to 48 hours. A secondary drainage must be resorted to if the liquid continues to accumulate. Irrigation with warm Dakin's solution, or " bipp " mixed with liquid paraffin, has given good results. Polyvalent anti-streptococcal serum has been applied with success in cases of empyema.

*Anesthetics* : (1) Local anesthesia : takes from 15 to 20 minutes to administer efficiently and often has to be supplemented with gas and oxygen.

(2) Chloroform and oxygen : has been used extensively with excellent results.

(3) Gas and oxygen : desperate cases stand gas and oxygen very well and recovery from the anesthetic takes place in a few minutes.

There follow tables showing in detail the results of operations, the stay at C. C. S's, the results obtained at the Base, in cases not operated on, etc.

The authors then draw the following conclusions, which complete the principles previously outlined.

(1) When intra-thoracic infection has not become localized, the chest should be closed and drained later, when necessary; primary drainage should be reserved, as a rule, for cavities of moderate size.

(2) The hemolytic streptococcus is one of the most dangerous organisms ; the gas bacillus is less dangerous, unless combined with other organisms.

(3) Cases of thoracotomy should, if possible, remain at the C. C. S. for 2 weeks or more after operation.

(4) Gas and oxygen is the best general anesthetic for chest cases.

(5) The use of morphine is of great value both in pre-operative and post-operative periods.

